# Do family characteristics explain a social gradient in overweight in early childhood?

**DOI:** 10.1093/eurpub/ckac129.581

**Published:** 2022-10-25

**Authors:** S Hoffmann, P Rattay, M Blume, C Hövener, S Schneider, I Moor, C Pischke, W Schüttig, F de Bock, J Spallek

**Affiliations:** 1 Brandenburg University of Technology, Department of Public Health, Senfenberg, Germany; 2 Robert Koch-Institute, Department of Epidemiology and Health Monitoring, Berlin, Germany; 3 Heidelberg University, Center for Preventive Medicine and Digital Health, Mannheim, Germany; 4 Martin Luther University Halle-Wittenberg, Institute of Medical Sociology, Halle, Germany; 5 Heinrich Heine University Düsseldorf, Institute of Medical Sociology, Düsseldorf, Germany; 6 Technical University of Munich, Department of Sport and Health Sciences, Munich, Germany; 7 Heinrich-Heine University Düsseldorf, Child Health Services Unit, Düsseldorf, Germany

## Abstract

**Background:**

Children's overweight is associated with many factors, including their living situation, in particular their family's socioeconomic position (SEP) and family characteristics. Research on the extent to which family characteristics account for a social gradient in overweight in early life is scarce. This study evaluated whether family characteristics explain SEP differences in the risk of overweight in early childhood.

**Methods:**

The study used baseline data of 3-6 year-old children (n = 1,116) from the intervention ‘Ene mene fit’ conducted at kindergartens in Baden-Württemberg, Germany. Data included overweight (body mass index > 90 percentile) and parents’ reports on their education and family characteristics associated with overweight (child consumes: sweets in front of TV, soft drinks; family joined time: outdoor, breakfast, sports; cooking; child sets table; role model). Model-based single mediation analyses decomposed the total effect of highest parental education on overweight into direct (unmediated) and indirect (mediated) effects (OR, 95% CI).

**Results:**

Girls and boys with low parental education had higher odds for overweight than children with high/medium education. Among boys, low education influenced the risk of overweight via indirect effects of i. ‘sweets consumption in front of TV’ (OR = 1.31, 1.05-1.59) and ii. ‘no joined sports’ (OR = 1.14, 1.00-1.44). The direct effect of low education only remained significant when ‘no joined sports’ was considered (OR = 2.19, 1.11-5.19). Among girls, family characteristics measured here did not explain SEP differences in overweight.

**Conclusions:**

The family characteristics ‘sweets consumption in front of TV’ and ‘no joined sports’ contribute to inequalities in overweight among boys, but not among girls. Therefore, more gender-sensitive research is needed to identify family risk and protective characteristics that explain health inequalities among both boys and girls.

